# Digital Health Revolution: Is it Time for Affordable Remote Monitoring for Parkinson’s Disease?

**DOI:** 10.3389/fneur.2015.00034

**Published:** 2015-02-26

**Authors:** Spyros Papapetropoulos, Georgia Mitsi, Alberto J. Espay

**Affiliations:** ^1^Department of Neurology, Miller School of Medicine, University of Miami, Boston, MA, USA; ^2^Massachusetts General Hospital, Boston, MA, USA; ^3^Apptomics LLC, Wellesley Hills, MA, USA; ^4^James J. and Joan A. Gardner Center for Parkinson’s Disease and Movement Disorders, University of Cincinnati Neuroscience Institute, Cincinnati, OH, USA

**Keywords:** Parkinson’s disease, health outcomes, digital health, disease management, remote monitoring

## Parkinson’s Disease is a Common Health Care Problem with Significant Social and Economic Implications

More than one million people in the US (five million people worldwide) are believed to have Parkinson’s disease, and its prevalence is expected to double by 2030 (1). The national annual economic burden for Parkinson’s disease in 2010 was estimated at more than $14.4B and is expected to grow substantially due to the increasing aging population (2). Disease prevalence is age-associated, with approximately 1–2% of the population being affected at 65 years (3). The cardinal clinical features of Parkinson’s disease are tremor, bradykinesia, rigidity, and postural instability (4). Parkinson’s disease significantly affects employability and activities of daily living, leading to a reduction in health-related quality of life and increases in morbidity and mortality (5, 6). Parkinson’s disease has a substantial impact on patients, caregivers, and the healthcare system, especially as the disease progresses and patients are less able to care for themselves (7).

While l-DOPA has resulted in significant improvement in quality of life and reduction in Parkinson’s disease-related mortality (8), a number of motor complications develop in approximately 50% of patients within 3–5 years (9). The most common l-DOPA-induced motor complications include motor fluctuations (e.g., wearing off, unpredictable “off–on” fluctuations) and dyskinesia can be more disabling than the motor symptoms for which treatment was initiated (10). Wearing off applies to rapid reduction in mobility; dyskinesia is expressed as dance-like, random involuntary movements, classified as chorea (typically when “on”) or dystonia (typically when “off”) (11). The pathogenesis of these changes is not well understood (12). These motor fluctuations and dyskinesia are managed with dose adjustment of l-DOPA and/or the co-administration of adjunctive therapies (dopamine agonists, MAO-B inhibitors, or amantadine). To design an appropriate management plan, specialized evaluation is needed. However, access to such care has become increasingly difficult. The most recent WHO atlas of resources for neurological disorders indicates that the lack of access to specialists varies from 0.03 to 4.84 per 100,000 population depending on geographic location (13).

## Pharmacotherapeutic Adjustments Require In-Office Clinical Evaluation and Interpretation of Patient-Reported Data

To appreciate the magnitude of motor deficits, a full neurological examination is required. The quantification of such deficits is often made using a clinical scale, the motor part of the Unified Parkinson’s Disease Rating Scale (UPDRS, or MDS-UPDRS) (14, 15). This scale provides clinicians with an opportunity to rate each motor domain (e.g., tremor, bradykinesia, rigidity, etc.) with an integer score ranging from 0 to 4 in graded severity. For the management of l-DOPA-induced motor complications, medical decisions are typically “empirical,” based on patient narratives about their experiences between visits or less frequently by a patient pen-and-paper diary. The latter requires patients’ input every 30 min in a complex time matrix. Reduced compliance and recall bias significantly limit the real-world utility of the diary in its current form (16). In clinical trial settings, it has been demonstrated that close patient monitoring and treatment “optimization” can lead to significant improvement of motor function and fluctuations even before administration of experimental interventions (17).

In summary, challenges related with current practices in management of Parkinson’s disease patients are: (1) infrequent visits and problematic access to specialists (2) inadequate monitoring of between-visit function, and (3) poor treatment optimization due to insufficient patient data. Patients may also delay or avoid chronic disease management services because they are costly, time-consuming, and difficult to come by as physicians’ time is increasingly constrained. As a result, patients’ functional state can be suboptimal and may lead to unnecessary evaluations in the emergency room and at times even costly interventions, all of which have the unintended consequence of increasing health resource utilization. In this scenario, user-friendly instruments for measuring motor function and monitoring treatment-induced motor complications in the home setting could revolutionize access to care and enhance treatment optimization with currently available drugs.

## Quantitative Portable Measurements are Easier to Administer and may Reduce the Need for In-Clinic Visits

Unlike clinical rating scales that utilize categorical ratings, objective symptom monitoring can quantify motor scores on a continuum, allowing for greater precision in recording subtle changes in Parkinson’s disease motor symptomatology (16). Several studies have demonstrated that remote monitoring systems and virtual visits improve the quality of care while minimizing direct and indirect healthcare costs (18–20). Introduction of simple, reliable, and sensitive objective measures to supplement the in-office clinical evaluation and extend it to a home environment has the potential to enhance management of Parkinson’s disease symptoms.

## Technology Holds the Promise of Better-Informed Medical Decisions during In-Clinic Visits

A recent review by Maetzler et al. discussed details of several promising wearable technologies, including which parameters (motor and non-motor disabilities) should be prioritized in assessment strategies in Parkinson’s disease. Therefore, a detailed review of different technologies and parameters is not included in this manuscript. The review concludes that the currently available technologies have not yet found their way into routine clinical assessment. The authors expect that in the near future, this is expected to drastically change and such techniques will help to overcome the drawbacks that are inherent to single or multiple “snapshot” assessments in current clinical practice and clinically oriented research (21). Technology holds the promise of better management of complicated PD patients but it is not expected to eliminate the need of regular office visits during which patients, caregivers, and physicians can interact face-to-face.

## The Changing Healthcare Market Landscape will Increase the Need for Remote Monitoring of Patients

An increasing proportion of patients and caregivers are connecting through their smartphones and tablets to a variety of resources to assist in their care. Insurers, pharmaceutical companies, and healthcare professionals are developing mobile apps and wearable sensors represent to support this need. In US, the shift toward “accountable” care will lead to wider adoption of remote monitoring solutions for chronic conditions. Juniper care predicted that by 2016, three million patients would be monitored remotely over cellular networks (22). Aetna, LifeWise, United Health, and Kaiser Permanente have included mobile apps as part of their strategy to improve customers’ experience. Pfizer, Boehringer Ingelheim, GlaxoSmithKline, and AstraZeneca have already invested time and effort in creating digital health solutions. Analysts predict that remote health is one of the fastest growing areas of Healthcare IT, and it is expected to save $36B by 2018 globally (23).

The Department of Veterans Affairs (VA) serves as an example of how government can advance important healthcare IT initiatives ahead of the private sector by its recent announcement to support telehealth by eliminating co-pays for patients taking advantage of virtual consultations (24). On the other end of the spectrum, healthcare professionals are connected with devices for a variety of uses and are already adopting and recommending apps to improve quality of care. Almost 90% of physicians would like their patients to monitor their health independently. There are already 75 FDA-cleared apps from a total of 13,000 claimed medical mobile applications (22).

## The Cost of Portable Measuring Devices is Decreasing: The Opportunities are there for the Taking

Enhancing the application of available treatments by leveraging rapidly growing technologies in a complex healthcare landscape offers both challenges and opportunities. Development of combined mobile applications and wearable sensor systems is an innovative concept that can have immediate healthcare implications. Such technologies could allow for remote monitoring/virtual visits, improved access, and reduced cost while improving the overall experience, especially for patients with chronic conditions. In a recent pilot clinical study of “virtual house calls” for Parkinson’s disease, remote visits saved participants on average 100 miles, and 3 h of travel (25). The development and validation of these tools takes a fraction of the time and cost compared to traditional drug development.

In conclusion, remote monitoring systems for Parkinson’s disease will support the patient, the caregiver, and the healthcare professional in their collaborative efforts for better care. Access is improved and even patients in distant areas can have their symptoms captured and transmitted to specialists remotely. The wealth of newly captured data in the natural ecological environment of patients has the capacity to enhance our understanding of a patient’s status and tailor his/her treatment according to specific motor function and complication profiles. Technology-driven optimization of therapy in Parkinson’s disease holds the promise of leading to faster access to care and more effective outcomes for individual patients while substantially decreasing healthcare costs compared to the current in-clinic visit model.

## Author Contributions

SP drafted the manuscript and was involved in its conception and critical review; GM was involved in the conception and critical review of the manuscript; AE was involved in the conception and critical review of the manuscript.

## Conflict of Interest Statement

Spyros Papapetropoulos is a full-time employee of Pfizer. Georgia Mitsi has an equity interest in Apptomics LLC. Alberto J. Espay is supported by the K23 career development award (NIMH, 1K23MH092735); has received grant support from CleveMed/Great Lakes Neurotechnologies, and the Michael J. Fox Foundation; personal compensation as a consultant/scientific advisory board member for Solvay (now Abbvie), Chelsea Therapeutics, TEVA, Impax, Merz, Pfizer, Solstice Neurosciences, Eli Lilly, and USWorldMeds; royalties from Lippincott Williams & Wilkins and Cambridge University Press; and honoraria from Novartis, UCB, TEVA, the American Academy of Neurology, and the Movement Disorders Society. He serves as Associate Editor for Frontiers in Neurology and the Journal of Clinical Movement Disorders, and on the editorial boards of Parkinsonism and Related Disorders and The European Neurological Journal.
